# A farewell from the founding Editor of Skin Health and Disease

**DOI:** 10.1002/ski2.221

**Published:** 2023-02-12

**Authors:** George W. M. Millington

**Affiliations:** ^1^ Dermatology Department Norfolk and Norwich University Hospital Norwich UK; ^2^ Norwich Medical School Norwich UK

1

After 6 years as the Editor in Chief of Clinical and Experimental Dermatology,^1^ one of the two established journals from the British Association of Dermatologists (BAD), in January 2020, I decided to take on a new challenge, launching a new open access (OA) dermatology journal, Skin Health and Disease (SHD).^2^ This was and still is the only purely OA journal published by the BAD.^2^, ^3^ From January 2020 to July 2020, I assembled a distinguished Editorial Advisory Board (EAB) and Editorial team (https://onlinelibrary.wiley.com/page/journal/2690442x/homepage/editorial-board). One of the first management tasks we had to grapple with was the issue of consent, which is a particularly important issue for OA journals.^4^

Our first manuscripts were published online in July 2020 and we published our first compete edition of the journal in March 2021 (https://onlinelibrary.wiley.com/toc/2690442x/2021/1/1). In my last edition as Editor, we published a special collection on Psychodermatology (https://onlinelibrary.wiley.com/toc/2690442x/2022/2/4)^5^, ^6^ and we have more special editions in the pipeline.^7^ Our biggest achievement so far was being listed on PUBMED Central after 21 months.

I have many people to thank. As SHD Editor, I have received the support of three BAD Presidents, Ruth Murphy, Tanya Bleiker and Mabs Chowdhury and two BAD CEO's, Marilyn Benham and Simon Morrison. The BAD publishing team, comprising Shehnaz Ahmed, John Caulfield, Jide Ibitoye and Laura Prescott, and the Wiley team of Sam Davies, Claire Bonnett, and Swaminathan Nagarajan have all been incredibly motivated at getting this project going and maintaining momentum. I have always been able to tap into the wisdom of my EAB and my editorial team have constantly been up to speed with processing manuscripts. Finally, I have to thank my good friend and former Deputy Editor, Ewan Langan. He and I are trading places after 3 years and he is your new Editor.

For updates on SHD content, follow me on Linkedin;

https://www.linkedin.com/in/dr-george-millington-50a74a14/.

## CONFLICT OF INTEREST STATEMENT

George W. M. Millington is the Deputy Editor in Chief of Skin Health and Disease.

## AUTHOR CONTRIBUTION

**George W. M. Millington**: Writing – original draft (Lead); Writing – review & editing (Lead).

## FUNDING INFORMATION

This editorial received no specific grant from any funding agency in the public, commercial, or not‐for‐profit sectors.

## ETHICS STATEMENT

Not applicable.

## Data Availability

Data sharing is not applicable to this article as no new data were created or analysed in this study.
